# Development and Validation of the Sharenting Behavior Scale Among Parents

**DOI:** 10.3390/bs16081416

**Published:** 2026-08-18

**Authors:** Şule Betül Tosuntaş, Mark D. Griffiths

**Affiliations:** 1 Department of Educational Sciences, Faculty of Education, Bursa Uludağ University, Bursa 16059, Turkey; 2 Nottingham Institute of Psychology, Nottingham Trent University, Nottingham NG1 4FQ, UK; mark.griffiths@ntu.ac.uk

**Keywords:** sharenting, social media, parents, scale development, psychometrics, exploratory factor analysis

## Abstract

Due to the proliferation of social media, sharenting has become a widespread parental behavior. Its prevalence, combined with its potential impact on children’s privacy and well-being, highlights the need for a parent-specific psychometric instrument. The present study aimed to develop and validate the Sharenting Behavior Scale (SBS) to assess the factors influencing sharenting behavior. A total of 1483 parents participated. Psychometric testing included construct validity, using exploratory and confirmatory factor analyses, and reliability assessment. Exploratory factor analysis yielded a 30-item, six-dimensional structure explaining 49.60% of the total variance. Confirmatory factor analysis validated this structure (robust CFI = 0.956, TLI = 0.950, RMSEA = 0.037, SRMR = 0.050). Convergent and discriminant validity were established, and concurrent validity was supported by positive associations with fear of missing out, social presence, and social media addiction. The total scale showed good internal consistency (Cronbach’s α = 0.86), with subscale alpha and McDonald’s omega coefficients ranging from 0.72 to 0.90. The scale further demonstrated full measurement invariance across gender, supporting comparisons between male and female parents. These findings indicate that the SBS is a valid and reliable instrument for assessing sharenting behavior among parents.

## 1. Introduction

In today’s digital world, the integration of social media into individuals’ everyday lives has brought about new online behaviors. Among these phenomena, ‘sharenting’ a term denoting parents’ practice of sharing information about their children on social media, has gained considerable attention from the wider public including researchers, media, and parents themselves (Blum-Ross & Livingstone, 2017; Brosch, 2018; Tosuntaş & Griffiths, 2024). As online spaces become repositories for personal stories and reflections, investigating sharenting holds significant importance in understanding the complex interplay between technology, parenthood, and social engagement. With the increase in social media use, sharenting has become a prevalent parental behavior. Notably, Nominet’s (2016) data indicate that parents share an average of nearly 1500 photos of their children before the age of five years. While existing research has offered valuable insights into this evolving behavior, there remains a crucial gap in the evaluation of sharenting behaviors, particularly within the framework of parental roles.

Research has consistently shown that a considerable number of parents are actively involved in sharing diverse content about their children on social media platforms, including images, videos, and various types of information (Aslan & Durmuş, 2020; Atwell et al., 2019; Fox & Hoy, 2019). These shared posts typically revolve around special occasions, celebrations, holidays, and milestones in their children’s lives (Kopecky et al., 2020). Surprisingly, empirical evidence suggests that the prevalence of sharenting behavior remains largely uninfluenced by parents’ level of awareness regarding potential risks, digital literacy, or competencies (Barnes & Potter, 2021; Ranzini et al., 2020). Parents rationalize their engagement in sharenting by emphasizing its role in memory preservation and sustaining connections with their social network, as well as serving as a platform for seeking parental advice and support (Aslan & Durmuş, 2020; Wen et al., 2022).

Sharenting behavior is perceived as a way for parents to validate their connection with others, despite potential online risks (Cino et al., 2020; Ömür & Uyar, 2022). Social media provides a distinctive platform for self-presentation, as observed in the typologies identified by Holiday et al. (2022), where parents portray themselves in polished, promotional, and/or intimate ways to convey relational dynamics, self-promotion, and parent–child bonds. For millennial parents, seeking social affirmation, receiving support, and demonstrating effective parenting have emerged as significant motives (Latipah et al., 2020; Ögel-Balaban, 2021; Udenze & Bode, 2020). Moreover, studies have highlighted the significance of impression management and seeking advice among mothers of younger children (Kumar & Schoenebeck, 2015; Marasli et al., 2016). As Brosch (2016) proposed, sharenting has evolved into a social behavior that fulfills parents’ needs for self-expression and social validation, reinforced by normative peer behavior around sharing (Walrave et al., 2022). Nonetheless, despite the many empirical studies, the precise psychosocial factors directly or indirectly influencing sharenting behavior remain unclear (Tosuntaş & Griffiths, 2024).

Sharenting poses several well-documented long-term risks for children, including the creation of a permanent and uncontrollable digital footprint, violations of privacy, exposure to data exploitation and commercial profiling, misuse of children’s images (e.g., digital kidnapping), and heightened vulnerability to cyberhate and future reputational harm (Blum-Ross & Livingstone, 2017; Wachs et al., 2021). Despite these risks, parents persist in such practices, leading to what is known as the ‘openness paradox’ (Chalklen & Anderson, 2017). This term captures the intricate balance between the immediate benefits and enduring risks of sharing personal information online and how parents navigate this delicate situation (Cino & Wartella, 2021). Notably, some individuals have transformed their sharenting activities into a profession, becoming social media influencers or bloggers specializing in parenting and family-related content.

The evolution of commercial sharenting into a profitable industry has become evident, with a minority of parents earning substantial sums for individual posts. Blum-Ross and Livingstone (2017) highlighted that professional sharents, like their non-professional counterparts, seek to chronicle their children’s memories, considering it an essential aspect of their parental responsibilities. Abidin (2015, 2017) emphasized that while children have a strong digital presence, their online engagements are yet to be formally recognized as labor, and the absence of a governing authority places them beyond the scope of child-labor regulations that traditionally apply to child stars. Collectively, the emergence of sharenting as a profession triggers significant concerns regarding the intersection of parenting and digital media, alongside the balance between safeguarding privacy and pursuing financial gain.

Children’s perspectives highlight that sharenting behaviors can potentially trigger conflicts between parents and their children, particularly concerning privacy and impression management. Images considered ‘cute’ by parents might lead to feelings of embarrassment and frustration among adolescents (Leaver, 2017). Nonetheless, when parents share moments of pride or happiness, sharenting appears more acceptable to children (Lipu & Siibak, 2019; Ouvrein & Verswijvel, 2019). Children tend to view sharenting positively when it aims to capture cherished memories. However, they express disapproval when parents exploit their images for their own self-presentation needs (Verswijvel et al., 2019). Interestingly, De Wolf (2020) found that over half of parents do not seek their children’s permission before sharing content about them.

Barassi (2020) challenged the commonly held idea propagated by the media that parents are largely inattentive to their children’s digital presence. Children also exhibit an increased awareness of the potential ramifications of sharenting. Wachs et al. (2021) concluded that sharenting was associated with elevated vulnerability to cyberhate among 5433 young participants (aged 12–16 years). This suggests that, in the long run, sharenting could yield adverse outcomes for children, even impacting their future job prospects (Lavorgna et al., 2022; Ouvrein & Verswijvel, 2019). To avoid this undesirable situation—often referred to as “digital tattoos” rather than “digital footprints” (Blum-Ross & Livingstone, 2017)—there is a growing consensus that children and parents should collaboratively set boundaries for sharenting behaviors (Lipu & Siibak, 2019; Ouvrein & Verswijvel, 2019; Sarkadi et al., 2020).

Theoretically, sharenting behavior can be understood through two complementary frameworks: the privacy/openness paradox and self-presentation theory. The privacy/openness paradox (Chalklen & Anderson, 2017) captures the tension between parents’ desire for social connectivity and their awareness of the risks associated with exposing children’s personal information in public digital spaces. Despite knowing the potential harms—ranging from data exploitation to cyberbullying—parents continue to engage in sharenting, often rationalizing this behavior through perceived social benefits (Barth & De Jong, 2017; Ní Bhroin et al., 2022). Self-presentation theory posits that parents use sharenting as a form of impression management, curating an idealized image of their family for online audiences (Holiday et al., 2022).

Together, these frameworks suggest that sharenting is a multidimensional behavior. The privacy/openness paradox foregrounds the cognitive and evaluative dimensions—parents’ awareness of risks and consequences, their conflicts over sharing, and their trust in platforms—while self-presentation theory foregrounds the motivational and social dimensions—the reinforcement, identity, and social-connection processes that drive sharing. This multidimensional conceptualization directly informed the domains that the Sharenting Behavior Scale (SBS) was designed to capture.

### The Present Study

With the increasing prevalence of sharenting, it has become an increasingly researched topic in recent years. The first psychometric study that aimed to assess sharenting behavior was conducted by Romero-Rodriguez et al. (2022), who developed the Sharenting Evaluation Scale (SES). However, the SES has several methodological limitations that constrain its utility. The original validation study used a broad non-parent sample: of the total 146 participants, only approximately 34% were parents, making the sample insufficiently representative of the target population. The small total sample size (N = 146) falls well below recommended minimum thresholds for stable exploratory and confirmatory factor analyses. Critically, the exploratory factor analysis (EFA) and confirmatory factor analysis (CFA) were conducted on the same participants rather than independent subsamples, precluding genuine cross-validation and inflating the risk of model overfitting.

Since the SES, a small number of further scale-development studies have been published. Wang et al. (2025) developed the Motivation of Sharenting Evaluation Scale (MSES) among Chinese parents, but it is confined to sharenting motivations (three factors, 10 items) and does not address risk awareness, conflict, or trust. Within the Turkish context, Cansızlar and Şahin (2024) developed the Sharenting Scale (SS), but it is a brief nine-item, two-factor instrument confined to parents’ self-presentation styles (a “polished” and an “intimate” profile) and does not capture the motivational, risk-awareness, conflict, or trust dimensions of sharenting. In addition, Kılıç et al. (2023) and Bayraktar and Ögel-Balaban (2026) provided Turkish adaptations of the SES; the latter two therefore inherit the aforementioned conceptual and structural constraints of the original SES. Across these efforts, no existing instrument offers a parent-specific, multidimensional assessment that jointly captures the motivational, awareness, conflict, and trust dimensions of sharenting. The SBS was developed to fill this gap.

These limitations highlight the need for a psychometric instrument designed specifically for parents, validated with adequate sample sizes and independent EFA and CFA subsamples, and capable of capturing the multidimensional nature of sharenting—including motivational, risk-awareness, conflict, and trust dimensions—within a rigorous psychometric framework. Sharenting decisions are uniquely embedded in parental roles and cultural contexts; an instrument developed and normed exclusively on parents is therefore more appropriate for theoretical modeling and applied research on this behavior.

Based on these two frameworks (the privacy/openness paradox and self-presentation theory), sharenting was expected to be multidimensional, spanning motivational drivers, emotional and social motives, risk and consequence awareness, interpersonal conflict, and trust in social media platforms. The specific factor structure was examined empirically through exploratory factor analysis and then tested with confirmatory factor analysis on an independent subsample. It was further expected that SBS scores would show positive associations with fear of missing out (FoMO), social presence, and social media addiction, constructs theoretically linked to excessive or compulsive social media engagement. Positive associations were also expected between SBS active-sharenting subscale scores and child-related posting frequency and daily social media time, because more engaged social media users would be expected to engage in more frequent sharenting.

## 2. Method

### 2.1. Participants

The participants of the study were parents residing in Türkiye who reported having at least one child. A total of 1483 participants provided e-consent and completed an online survey. Age was assessed using five ordinal categories (18–25 years, 26–35 years, 36–45 years, 46–55 years, and 56 years or older). Participant demographic characteristics are presented in Table 1.

Regarding gender, 747 participants identified as female (50.4%) and 736 as male (49.6%). Regarding age distribution of the total 1483 participants, 10 participants were aged 18–25 years (0.7%), 187 participants aged 26–35 years (12.6%), 619 participants aged 36–45 years (41.7%), 447 participants aged 46–55 years (30.1%), and 220 participants aged 56 years or older (14.8%). The majority of participants were married (91.8%), followed by single individuals (6.8%), and those in a relationship (0.5%). Thirteen participants chose not to disclose their marital status. Among the participants, 669 indicated having one child (45.1%), 630 had two children (42.5%), 154 had three children (10.4%), and 30 had four or more children (2.0%).

### 2.2. Procedure and Questionnaire Development

Items were generated through a comprehensive review of existing literature on sharenting, privacy behavior, and parental social media use (Tosuntaş & Griffiths, 2024). An initial pool of 73 items was formulated to span the dimensions identified in the literature review, including motivational drivers, emotional and social motives, risk and consequence awareness, interpersonal conflict, and trust in social media platforms. The items were reviewed by a panel of experts in clinical psychology, measurement, and digital media. Expert feedback was used to refine item wording, eliminate redundancies, and ensure face validity. Although formal Content Validity Indices (CVI) were not computed in this phase, expert-based item review to refine item content is an accepted practice in early-stage scale development (Lynn, 1986; Polit & Beck, 2006).

A five-point response scale from 1 (*definitely disagree*) to 5 (*definitely agree*) was used for all items. A five-point format was selected because it offers a neutral midpoint and provides reliability and validity comparable to formats with more categories, while minimizing participant burden. Scales with fewer than five categories tend to show lower psychometric quality, whereas gains beyond five to seven categories are marginal (Preston & Colman, 2000).

An online survey was hosted on the *Qualtrics* platform and distributed to parents through institutional and professional networks across Türkiye. The survey presented the measures in a fixed order: demographic and social-media-use questions first, followed by the SBS, and then the psychometric scales assessing FoMO, social presence, and social media addiction. The social media use and sharenting-practice questions (e.g., platforms used, daily time online, posting frequency) were single-item descriptive measures, for which internal consistency estimates are not applicable. Completing the survey took approximately 20 min. Individuals who reported that they did not have any children were excluded from the study. Online informed consent was obtained from all participants. The data collection period spanned from June to July 2023. The study obtained ethical approval from the first author’s university research ethics committee and was conducted in accordance with the principles outlined in the Helsinki Declaration.

### 2.3. Measures

*Demographic and sharenting variables:* Participants were asked demographic questions regarding gender, age range, marital status, and number of children. Detailed statistics are presented in Table 1. Furthermore, participants were asked about their use of social media applications, the amount of daily time spent on the internet and social media, the frequency of sharing content related to their children, the topics of their child-related posts, who the child was within the posts when sharing, and the types of content they shared (multiple selections were possible). Detailed statistics are presented in Table 2.

*Sharenting Behavior Scale (SBS):* The SBS was developed by the present authors with the aim of assessing parents’ sharenting behavior. The initial SBS had 73 items. Items (e.g., *“I’m careful not to share sensitive information or images of my child on social media”*) are rated on a five-point scale from 1 (*definitely disagree*) to 5 (*definitely agree*). A higher score indicates a higher level of sharenting behavior. The detailed statistical analysis of the SBS’s psychometric properties is presented in the Results section. The final version of the SBS is presented in Appendix A.

*Fear of Missing Out (FoMO):* FoMO was assessed using the Fear of Missing Out Scale, originally developed by Wegmann et al. (2017) and adapted into Turkish by Balta et al. (2020). The scale comprises 12 items (e.g., *“I am continuously online in order not to miss out on anything”*) rated on a five-point Likert scale from 1 (*never*) to 5 (*always*). The Turkish form was used for the first time in the adaptation study, where CFA supported the two-factor structure (χ^2^/df = 3.80, root mean square error of approximation [RMSEA] = 0.08 [90% confidence interval [CI]: 0.07, 0.09], standardized root mean square residual [SRMR] = 0.02, comparative fit index [CFI] = 0.93), with subscale internal consistencies of 0.78 and 0.85 (Balta et al., 2020). In the present sample, Cronbach’s α was 0.75 (trait), 0.83 (state), and 0.85 (total). Higher scores indicate greater FoMO.

*Social Presence:* Social presence was assessed using the Social Presence Scale, originally developed by Gao et al. (2017) and adapted into Turkish by Kırcaburun and Griffiths (2019). The scale comprises five items (e.g., *“There is a sense of human contact in Instagram”*) rated on a seven-point Likert scale from 1 (*absolutely not true*) to 7 (*absolutely true*). The Turkish form was used for the first time in the adaptation study, where CFA supported the unidimensional structure (χ^2^/df = 0.71, RMSEA = 0.00 [90% CI: 0.00, 0.06], SRMR = 0.02, CFI = 1.00), with an internal consistency of 0.74 (Kırcaburun & Griffiths, 2019). In the present sample, Cronbach’s α was 0.82. Higher scores indicate greater perceived social presence in social media use.

*Bergen Social Media Addiction Scale (BSMAS):* Social media addiction was assessed using the Bergen Social Media Addiction Scale, developed by Andreassen et al. (2016) as an adaptation of the Bergen Facebook Addiction Scale (Andreassen et al., 2012), with a validated Turkish translation by Tosuntaş et al. (2024). It consists of six items (e.g., *“How often during the last year have you felt an urge to use social media more and more?”*) rated on a five-point Likert scale ranging from 1 (*never*) to 5 (*always*). Total scores can range from 6 to 30, with scores of 19 or above signaling risk for social media addiction. The Turkish form was used for the first time in the adaptation study, where CFA showed good fit (χ^2^/df = 2.18, RMSEA = 0.04 [90% CI: 0.01, 0.07], SRMR = 0.02, CFI = 0.99), with standardized loadings from 0.46 to 0.78 and an internal consistency of 0.81. In the present sample, Cronbach’s α was 0.88.

### 2.4. Statistical Analysis

All statistical analyses were conducted in *R* version 4.6.0 (R Core Team, 2026) using the *psych* (version 2.6.5; Revelle, 2026) and *lavaan* (version 0.6-21; Rosseel, 2012) packages. Exploratory and confirmatory factor analyses, parallel analysis, reliability estimation, and validity correlations were computed with *psych*; the multigroup six-factor CFA for measurement invariance was estimated with *lavaan*.

Of the 1483 participants, 1368 provided complete responses on the SBS items and were retained for the complete-case psychometric analyses (listwise deletion). This complete-case sample was randomly partitioned into two independent halves (seed = 2023): an EFA subsample (*n* = 684) and a CFA subsample (*n* = 684). EFA and CFA were conducted on separate subsamples to provide independent cross-validation. Reliability, inter-factor correlations, and validity correlations were computed on the full complete-case sample (N = 1368) unless otherwise stated.

Item analysis included computation of item means, standard deviations, skewness, and excess kurtosis. Prior to factor analysis, an initial item-analysis screening was conducted to remove items with weak discrimination, a standard preliminary step in scale development (DeVellis, 2017). Items with item-total correlations below 0.30 and items that failed to differentiate the upper and lower 27% groups were eliminated, so that the subsequent exploratory factor analysis was performed on a set of items already demonstrating adequate discrimination. This two-stage approach reduced the likelihood that poorly performing items distort the emerging factor structure. In the subsequent EFA, items with factor loadings below |0.40| on their primary factor, loadings exceeding |0.40| on two or more factors with a gap between the highest and second-highest loading of less than 0.10, or problematic item statistics were candidates for elimination.

To determine the number of factors to retain, Horn’s parallel analysis was conducted (Horn, 1965) on the EFA subsample using FA-based eigenvalues of the reduced correlation matrix (squared multiple correlations on the diagonal), with 1000 simulated random datasets (seed = 42). Factors were retained when the observed FA eigenvalue exceeded the mean of the corresponding random eigenvalue distribution, the recommended criterion for common-factor analyses (Horn, 1965; Fabrigar et al., 1999). Maximum likelihood (ML) EFA with oblimin rotation was then conducted on the EFA subsample (*n* = 684) to produce a latent-variable solution appropriate for scale development (Costello & Osborne, 2005; Fabrigar et al., 1999). ML EFA, unlike PCA, estimates only the common-factor variance, yielding communalities and factor loadings that better reflect the latent structure underlying the items.

Sampling adequacy was assessed via the Kaiser–Meyer–Olkin (KMO) index and Bartlett’s test of sphericity. Prior to CFA, multivariate normality was assessed using Mardia’s coefficients. Both multivariate skewness (*b*_1p_ = 125.42, *p* < 0.001) and kurtosis (*b*_2p_ = 1135.61, *z* = 52.41, *p* < 0.001) departed significantly from normality, consistent with the pronounced floor effects observed for several items (Table 3). Consequently, CFA was conducted on the independent CFA subsample (*n* = 684) to evaluate the hypothesized six-factor model using robust maximum likelihood estimation (MLR), which provides a Satorra–Bentler scaled test statistic and robust standard errors appropriate for non-normal continuous indicators (Satorra & Bentler, 1994).

Competing CFA models (one-factor, four-factor, six-factor correlated, and second-order) were compared using the scaled χ^2^, degrees of freedom, and robust CFI, TLI (Tucker–Lewis Index), RMSEA, and SRMR. A second-order model was additionally specified to test whether the six correlated factors could be accounted for by a single general sharenting factor, which would justify interpreting a total SBS score and clarify the standing of each subscale within the broader construct. Model fit was evaluated against conventional criteria: CFI ≥ 0.95, RMSEA ≤ 0.06, and SRMR ≤ 0.08 indicate excellent fit; CFI ≥ 0.90 and RMSEA ≤ 0.08 indicate acceptable fit (Hu & Bentler, 1999; Kline, 2005).

Reliability was assessed using Cronbach’s alpha and McDonald’s omega on the full complete-case sample (N = 1368). Although the two coefficients rely on different assumptions, with alpha assuming tau-equivalence and omega not requiring it, both are reported to facilitate comparison with prior studies because alpha remains the most widely reported reliability index while omega provides a more accurate estimate when tau-equivalence does not hold (McNeish, 2018). Composite reliability (CR), which indexes the internal consistency of indicators loading on the same factor, and average variance extracted (AVE), which reflects the proportion of indicator variance captured by the latent factor, were computed from CFA standardized estimates (CFA subsample, *n* = 684). Item 1 was reverse-scored throughout.

Because the Awareness and Consequences subscale (F4) loaded negatively on the general second-order factor, its items were reverse-scored when computing the total SBS score, while retaining their original direction in subscale-level analyses. All scale and subscale scores, for both the SBS and the criterion measures, were computed as item means, so that scores remained on the original response metric. Concurrent validity was examined by computing Pearson correlations between SBS subscale and total scores and scores on FoMO, social presence, and social media addiction. Criterion-related validity was assessed using correlations between SBS scores and child-related posting frequency (reverse-scored) and daily social media time.

Discriminant validity was assessed using the Fornell–Larcker criterion (Fornell & Larcker, 1981) and the heterotrait–monotrait (HTMT) ratio of correlations, with 95% confidence intervals obtained through bootstrapping (Henseler et al., 2015). Measurement invariance across gender was examined in a configural → metric → scalar sequence using *lavaan* in *R* (Cheung & Rensvold, 2002); the ΔCFI ≤ 0.01 criterion was also applied. Gender was selected as the grouping variable for measurement invariance for three reasons. First, parental gender is the most consistently documented source of variation in sharenting, with mothers and fathers differing in both frequency and motivation (Fox & Hoy, 2019; Kumar & Schoenebeck, 2015). Establishing invariance across gender is therefore a prerequisite for the meaningful gender comparisons that the SBS is likely to be used for. Second, gender provided two large and approximately balanced groups (682 males, 681 females), which is well suited to multigroup analysis. Other demographic variables were less appropriate: age was assessed only in ordinal categories, and marital status was highly skewed (over 90% married), neither of which yielded balanced groups for invariance testing.

## 3. Results

Within the scope of the study, analyses were conducted with the data collected from 1483 participants. Participants used a variety of social media platforms, most commonly *WhatsApp* (91.7%), *Instagram* (66.1%), and *YouTube* (61.6%), while only a small minority (1.6%) reported using no social media. Most participants spent one to three hours per day on social media (55.8%), and a substantial proportion reported sharing little or no content about their children. Nearly half (49.5%) never posted child-related content, whereas only 3.2% did so on most days. When parents did share, the most common topics were special occasions (70.2%), family holidays and travel (41.9%), and their child’s achievements (38.3%). Most posts featured children together with family members (83.5%), and photographs were by far the most frequently shared content type (87.5%), followed by stories (26.6%) and videos (18.5%). A detailed breakdown of social media use and sharenting variables is presented in Table 2.

Regarding the context of sharing, participants noted that the majority of posts featured their children together with family members (83.5%), followed by posts featuring their children alone (30.2%), with friends (27.4%), and in group settings (11.7%). The most prevalent types of content shared were photos (87.5%), stories (26.6%), videos (18.5%), and text-based posts (10.9%).

### 3.1. Item Analysis

The development of the SBS proceeded in two stages of item reduction. In the first stage, the 73 initial items were screened for discrimination using item–total correlations, item–rest correlations, and comparisons between the upper and lower 27% groups. Items that correlated weakly with the overall scale or that failed to differentiate between the upper and lower groups were flagged as poor discriminators and removed from further consideration.

In the second stage, the retained items were submitted to exploratory factor analysis (Section 3.2). Items were eliminated iteratively when they loaded below the |0.40| threshold on their primary factor, loaded at or above |0.40|on two or more factors with a difference of less than |0.10| between the highest and second-highest loadings, or were otherwise inconsistent with a clear simple structure. Items were removed one at a time because removing an item redistributes the shared variance and can alter the loadings of the remaining items. The analysis was repeated after each removal until a clean six-factor structure was obtained, in which each retained item loaded on a single factor. This process reduced the pool from 73 to the final 30 items, distributed across the six subscales.

Descriptive statistics for the 30 retained items are presented in Table 3. Most items showed positive skew and moderate excess kurtosis, indicating floor effects consistent with the low base rate of overt sharenting behaviors in this sample, whereas the F4 (Awareness and Consequences) items showed the highest means, reflecting strong endorsement of risk awareness.

### 3.2. Exploratory Factor Analysis

Prior to EFA, sampling adequacy was assessed on the EFA subsample (*n* = 684). KMO = 0.939, indicating excellent sampling adequacy; Bartlett’s χ^2^ (435) = 8609.60, *p* < 0.001, confirming suitability for factor analysis. The number of factors was determined using factor-analytic parallel analysis (Horn, 1965), a method recommended over the Kaiser eigenvalue-greater-than-one rule because the latter tends to over-extract factors (Costello & Osborne, 2005). Horn’s parallel analysis using FA-based eigenvalues (1000 random datasets, seed = 42, mean criterion) supported retaining six factors: the observed FA eigenvalues for Factor 1 to Factor 6 all exceeded their mean random counterparts (Real FA EVs: 8.64, 2.42, 1.41, 0.89, 0.60, 0.49 vs. mean random: 0.46, 0.40, 0.36, 0.32, 0.29, 0.26), whereas Factor 7 did not (real = 0.18, mean random = 0.23). Initial eigenvalues for the six factors under the Kaiser (>1) criterion were: 9.16, 2.97, 2.08, 1.48, 1.11, and 1.08. For transparency, a PCA-style raw-eigenvalue parallel analysis indicated four factors; the FA-based method appropriate for common-factor analysis indicates six.

Multiple retention criteria therefore converged on six factors: the FA-based parallel analysis, the Kaiser eigenvalue > 1 rule, the CFA model comparison (ΔCFI = 0.189 favoring six factors over four; Section 3.3), and the theoretical framework each independently supported a six-factor solution (Costello & Osborne, 2005; Fabrigar et al., 1999).

ML EFA with oblimin rotation was conducted on the EFA subsample (*n* = 684). The six factors jointly accounted for 49.60% of the variance (unrotated ML extraction; Table 4). All 30 items loaded predominantly on their theoretically hypothesized factor (primary loadings ranging from |0.34| to |0.83|; Table 5). Beyond the |0.40| retention criterion, the solution showed a clean simple structure: no item had a secondary loading exceeding |0.30|, with the single exception of Item 7 (F1 primary = 0.47; F3 cross-loading = 0.29; gap = 0.18). One item showed a primary loading marginally below the 0.40 threshold under ML: Item 4 (F1, λ = 0.344). Item 4 (*“I like to see how my parenting practices compare to other parents on social media”*) captures social comparison, a well-documented motivational driver of sharenting through which parents evaluate their own parenting against others (Tosuntaş & Griffiths, 2024).

Although its EFA loading (0.344) fell marginally below the 0.40 criterion, the item was retained because social comparison represents a conceptually distinct facet of the reinforcement processes underlying F1 that the remaining items do not fully capture, and its CFA standardized estimate was adequate (β = 0.631). Removing it would have narrowed the construct coverage of the subscale. Item 1 (*“I’m careful not to share sensitive information”*) is the only reverse-worded item on F1. It loaded negatively in the EFA (−0.558), reflecting its protective wording, and was reverse-scored prior to the CFA, after which all seven F1 items loaded positively (0.516–0.724).

### 3.3. Confirmatory Factor Analysis

CFA was conducted on the independent CFA subsample (*n* = 684) to evaluate the six-factor correlated model and to compare it against competing models. Table 6 presents fit indices for the four competing models. The six-factor correlated model showed excellent fit on the independent CFA subsample: scaled χ^2^(390) = 708.06, robust CFI = 0.956, TLI = 0.950, RMSEA = 0.037 (90% CI [0.032, 0.041]), SRMR = 0.050, replicating the factor structure identified in the EFA. This model substantially outperformed the one-factor model (ΔCFI = 0.292) and the four-factor model (ΔCFI = 0.189), providing empirical support for the six-factor structure, converging with the FA-based parallel analysis and theoretical expectations. The higher-order (second-order) model showed acceptable fit (robust CFI = 0.934, RMSEA = 0.044) and is discussed further in Section 3.4.4.

To complement the global fit indices, local fit was examined through standardized residual correlations and modification indices. Residuals were uniformly small (M = 0.040, maximum = 0.153), with only 25 of 435 residual correlations (5.7%) exceeding 0.10, indicating no localized areas of substantial misfit. Modification indices did not suggest any substantively meaningful re-specification. The largest values corresponded to cross-loadings that would compromise the simple structure of the scale and were therefore not incorporated.

### 3.4. Validity

#### 3.4.1. Within-Scale Convergent and Discriminant Validity

Convergent validity within the SBS was evaluated using CR and AVE (Table 7). All CR values exceeded the 0.70 threshold (range: 0.72–0.89), indicating adequate construct reliability. AVE values ranged from 0.35 to 0.58. Four subscales showed AVE below the conventional 0.50 criterion: F4 Awareness and Consequences (0.35), F1 Reinforcements and Awareness (0.43), F5 Conflict (0.43), and F3 Social and Emotional Motives (0.49). The corresponding CR values for all four exceeded 0.70, indicating that the constructs explain more variance than measurement error. Hair et al. (2018) noted that AVE below 0.50 is acceptable when CR is high, particularly for affective or behavioral constructs assessed with heterogeneous item sets.

Discriminant validity was assessed using both the Fornell–Larcker criterion and the heterotrait–monotrait (HTMT) ratio of correlations (Henseler et al., 2015). The square roots of AVE were: F1 = 0.66, F2 = 0.76, F3 = 0.70, F4 = 0.59, F5 = 0.66, F6 = 0.72 (Table 7). Using the Fornell–Larcker criterion, most inter-subscale correlations (Table 8) were lower than the corresponding AVE square roots. However, the F1–F3 latent correlation approached the F3 AVE square root, a boundary condition that the Fornell–Larcker criterion detects with limited sensitivity (Henseler et al., 2015). The HTMT ratios provided a more robust test (Table 9). All ratios fell below the conservative 0.85 threshold, with the highest—F1–F3—at 0.75 (95% bootstrap CI [0.70, 0.80]), its upper bound remaining below 0.85. Discriminant validity was therefore supported for all six subscales.

#### 3.4.2. Concurrent Validity

To assess concurrent validity, Pearson correlations were computed between SBS total and subscale scores and three theoretically related external constructs: FoMO, social presence, and social media addiction (Table 10). SBS total scores correlated positively and significantly with FoMO (*r* = 0.42, *p* < 0.001), social presence (*r* = 0.37, *p* < 0.001), and social media addiction (*r* = 0.31, *p* < 0.001). The active-sharenting subscales (F1, F2, F3) showed similar positive associations with all three external constructs (*r*-values = 0.24–0.43). Notably, F4 (Awareness and Consequences) showed small but significant negative associations with FoMO (*r* = −0.14) and social media addiction (*r* = −0.10), and near-zero correlation with social presence (*r* = −0.06), consistent with F4 assessing protective cognition inversely related to addictive social media use.

#### 3.4.3. Criterion-Related Validity

Criterion-related validity was assessed via correlations with child-related posting frequency and daily social media time, shown in Table 10. SBS total scores showed a moderate positive correlation with posting frequency (*r* = 0.43, *p* < 0.001), consistent with theoretical expectations (i.e., parents who posted more frequently about their children scored higher on the SBS). Daily social media time also showed a small positive association with SBS total (*r* = 0.16, *p* < 0.001). Among subscales, F2 (Motives and Risk Awareness) showed the strongest positive association with posting frequency (*r* = 0.45), followed by F3 (*r* = 0.35) and F6 (*r* = 0.31). F4 (Awareness and Consequences) showed a small but significant negative association with posting frequency (*r* = −0.17), reflecting that parents who are more aware of risks tend to post less frequently, consistent with F4 capturing protective cognition that is behaviorally distinct from active sharenting.

#### 3.4.4. Higher-Order Factor Structure

**Figure 1:**
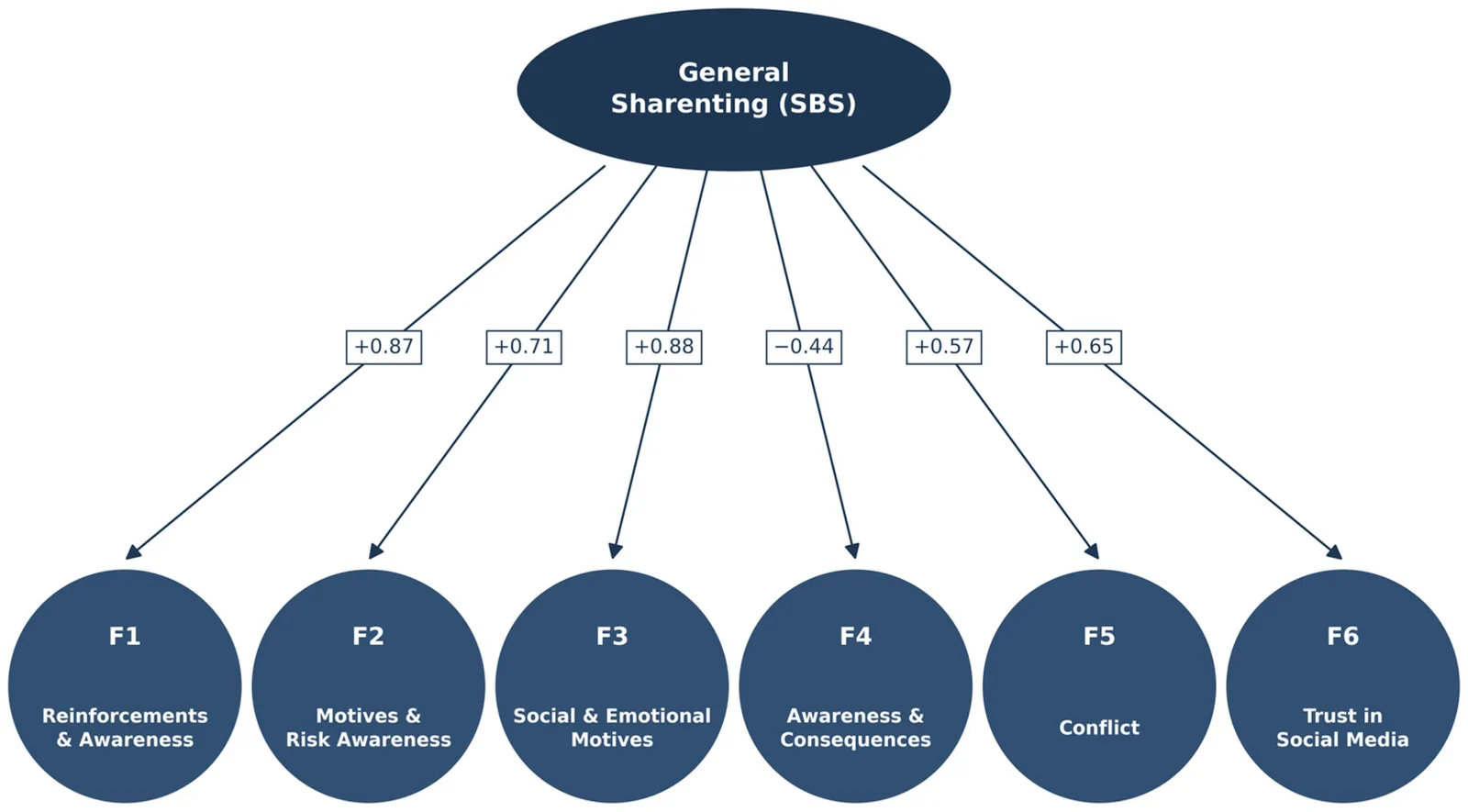
Higher-order confirmatory factor model of the Sharenting Behavior Scale.

A second-order CFA was fitted with the six first-order factors loading on a single general Sharenting (SBS) factor (CFA subsample, *n* = 684). The higher-order model showed acceptable fit: scaled χ^2^(399) = 874.80, robust CFI = 0.934, RMSEA = 0.044 (Table 6; Figure 1). The standardized second-order loadings were positive for the five active-sharenting factors—F1 (β = 0.874), F2 (β = 0.713), F3 (β = 0.879), F5 (β = 0.566), and F6 (β = 0.654)—and negative for F4 (β = −0.444; all *p* < 0.001). The negative loading of F4 indicated that as the general active-sharenting tendency increased, awareness of consequences tended to decrease, consistent with F4 assessing protective cognition that is conceptually distinct from pro-sharenting motives (see Section 4.1).

### 3.5. Reliability

The Cronbach’s alpha internal consistency coefficient for the 30-item SBS was 0.91. Internal consistency coefficients for the six subscales ranged from 0.72 to 0.89 (Table 7). McDonald’s omega ranged from 0.72 to 0.90, consistent with the alpha values. Composite reliability was also satisfactory (range: 0.70–0.90), supporting the internal consistency of the measurement model. The SBS can be used both at the subscale level and as a total score. As a multidimensional instrument, its six subscales capture distinct facets of sharenting behavior and can be analyzed separately, while the total score provides an overall index.

### 3.6. Measurement Invariance Across Gender

Measurement invariance of the SBS across gender was examined using a multigroup CFA sequence (configural → metric → scalar) in *R* (*lavaan* package), with 682 males and 681 females (total N = 1363; five participants with missing gender data were excluded from the invariance analysis). Table 11 presents fit indices for each model. The configural model, which allows all parameters to vary freely across groups, showed excellent fit: χ^2^(780) = 1585.55, CFI = 0.951, TLI = 0.945, RMSEA = 0.039, confirming that the six-factor structure holds in both gender groups separately. Constraining factor loadings to equality across groups (metric model) yielded χ^2^(804) = 1631.25, CFI = 0.949, TLI = 0.945, RMSEA = 0.039. The decrease in fit relative to the configural model was ΔCFI = 0.001, well within the Cheung and Rensvold (2002) criterion of ≤0.01, supporting metric invariance. Further constraining item intercepts to equality (scalar model) yielded χ^2^(828) = 1744.61, CFI = 0.944, TLI = 0.941, RMSEA = 0.040; ΔCFI = 0.005, again satisfying the ≤0.01 criterion and supporting scalar invariance. Although chi-square difference tests were statistically significant (metric vs. configural: Δχ^2^(24) = 45.70, *p* = 0.005; scalar vs. metric: Δχ^2^(24) = 113.36, *p* < 0.001), chi-square difference tests are known to be oversensitive when the sample size is large; invariance decisions therefore rest on the ΔCFI criterion (Cheung & Rensvold, 2002). Full scalar invariance supported the use of the SBS for comparing factor loadings, item intercepts, and latent means across male and female parents.

### 3.7. Age-Related Differences

To address whether sharenting varied with parental age, one-way ANOVAs compared the five age groups on child-related posting frequency and on SBS total and subscale scores (Table 12). Parental age was significantly associated with posting frequency, F(4, 1394) = 9.67, *p* < 0.001, as well as with SBS total scores and all subscales except F4 (all *p*-values < 0.01), although the effect sizes were small (*η*^2^ = 0.010–0.027). The pattern was non-monotonic rather than a simple decline with age. Posting frequency and the active-sharenting subscales (F1, F2, F3, F5, F6) were lowest among parents aged 36–55 years and comparatively higher among younger (26–35 years) and older (56+ years) parents. Post-hoc comparisons (Tukey HSD, corroborated by the Games–Howell procedure for unequal variances) confirmed this pattern for posting frequency, with the 46–55 years group scoring significantly lower than the 26–35 years group (*p* < 0.001) and the 56+ years group scoring significantly higher than the 46–55 years group (*p* < 0.001). In contrast, F4 (Awareness and Consequences) did not differ significantly across age groups (F[4, 1403] = 1.59, *p* = 0.17, *η*^2^ = 0.005), indicating that risk awareness was endorsed at comparable levels regardless of parental age. Because the youngest age group was small (*n* = 10), estimates for this group should be interpreted with caution.

## 4. Discussion

### 4.1. Reliability and Validity of the SBS

The aim of the present study was to develop and validate the SBS as a comprehensive psychometric instrument for assessing the multidimensional nature of parental sharenting behavior. The split-half cross-validation design—with EFA conducted on one independent half-sample (*n* = 684) and CFA on the other half (*n* = 684)—provides strong methodological support for the replicability of the six-factor structure. The six-factor correlated CFA model showed excellent fit on the held-out CFA subsample (robust CFI = 0.956, RMSEA = 0.037, SRMR = 0.050), substantially outperforming both the one-factor (ΔCFI = 0.292) and four-factor (ΔCFI = 0.189) alternatives.

Multiple factor-retention criteria independently supported the six-factor solution: the FA-based parallel analysis (six FA eigenvalues exceeding mean random), the CFA model comparison (ΔCFI = 0.189 over a four-factor model), and the theoretical framework each converged on six factors (Costello & Osborne, 2005; Fabrigar et al., 1999). The six-factor structure should nevertheless be further cross-validated using independent samples.

The higher-order model yielded two insights. First, the five active-sharenting factors (F1, F2, F3, F5, F6) loaded strongly and positively on a single general factor, indicating that these dimensions share a common underlying sharenting tendency and supporting the interpretation of a total SBS score as a summary index. Second, F4 (Awareness and Consequences) loaded in the opposite direction to the active-sharenting factors, and it showed small negative associations with posting frequency (*r* = −0.17) and social media addiction (*r* = −0.10). This pattern indicated that F4 captured a protective cognitive dimension—parents’ awareness of the legal, privacy, and reputational risks of sharenting—that is conceptually distinct from the motivational and social drivers of active sharing.

Theoretically, this maps onto the privacy-calculus literature (Barth & De Jong, 2017). Risk awareness does not straightforwardly prevent sharenting but constitutes a distinct psychological dimension that is best assessed and interpreted at the subscale level. Consistent with the present findings, a recent Italian study (Ferrara et al., 2024) reported that parents who shared less frequently were more likely to perceive excessive sharenting as neglectful, whereas frequent sharers showed lower risk perception. This parallels the negative association between F4 (awareness) and posting frequency observed here, providing external, independent support for the interpretation of F4 as a protective cognitive dimension.

The ‘reinforcements and awareness’ subdimension provides a nuanced understanding of the drivers underlying parents’ sharenting decisions about their children online, offering insight into the interplay between the desire for validation, social recognition, and the consideration of potential consequences for a child’s privacy. Consistent with the literature, sharenting has been characterized as a behavior that serves as a means of self-expression and social validation (Brosch, 2016; Cino et al., 2020). In particular, the efforts of young parents to establish their identity as caregivers play a motivating role in promoting sharenting behavior (Kumar & Schoenebeck, 2015; Latipah et al., 2020; Ögel-Balaban, 2021; Udenze & Bode, 2020).

The ‘motives and risk awareness’ subdimension reflects the relationship between parents’ reasons for sharenting and their awareness of potential risks, embodying the essence of the privacy paradox. It captures the privacy or openness paradox demonstrated by parents’ persistence in sharenting even though they are aware of potential long-term risks (Chalklen & Anderson, 2017). It also reflects the emotional facets of sharenting, highlighting the gratification parents derive from sharing feelings of pride, happiness, and excitement online. Consistent with this, Walrave et al. (2022) concluded that parents often share information about their children out of a sense of pride. These insights illustrate the delicate balance between parents’ motives, risk awareness, and the emotional rewards tied to sharenting.

The ‘social and emotional motives’ subdimension reflects how social media connects parents and offers emotional reinforcement. It extends beyond information sharing, highlighting the role of sharenting in fostering connections and mutual support, including the empathetic aspect of sharing personal stories for mutual understanding. By framing social media as a source of emotional support, the items emphasize online platforms as spaces for comfort, validation, and advice. Therefore, this subdimension recognizes the role of sharenting in nurturing connection and emotional support in modern parenting, where justifications often include the benefits of parental advice, support, and shared experience (Aslan & Durmuş, 2020; Wen et al., 2022).

The ‘awareness and consequences’ subdimension reflects parents’ recognition of the potential outcomes of sharing their children’s information on social media, including legal implications, future digital footprints, and broader consequences, as well as their conscious evaluation of both the benefits and drawbacks of sharenting. Numerous studies have indicated that parents often share their children’s personally identifiable information for reasons such as marketing, without fully considering the impact on their children’s digital footprints (Cino & Wartella, 2021; Fox & Hoy, 2019; Marasli et al., 2016). At the same time, some parents express concern about potential negative outcomes, including the misuse of their children’s images (Cino & Wartella, 2021).

The ‘conflict’ subdimension reflects the challenges parents face when engaging in sharenting, encompassing both internal and external conflicts arising from differing viewpoints, negative feedback, external pressure, and familial disagreement. Studies that have considered children’s perspectives demonstrate that children expect their parents to respect their boundaries and that conflict arises when these boundaries are disregarded (De Wolf, 2020; Ouvrein & Verswijvel, 2019; Verswijvel et al., 2019). Such conflicts related to sharenting commonly occur among family members (Ouvrein & Verswijvel, 2021), and parents also face dilemmas about sharing images of other children, such as those of extended family members (Cino & Vandini, 2020a, 2020b).

Finally, the ‘trust in social media’ subdimension reflects parents’ trust in the security and protective measures provided by social media platforms when sharing information about their children, contributing to an understanding of how parents perceive the safety and privacy dimensions of sharenting. Research suggests that parents’ digital competencies do not necessarily reduce sharenting; indeed, more proficient parents may share more frequently (Ní Bhroin et al., 2022). Lavorgna et al. (2022) emphasized the importance of examining the regulation and moderation policies of major social media companies, because such policies shape the boundaries of acceptable content, and argued that platform design should account for the actual impact of content moderation on potentially harmful behaviors. More recent work on commercial sharenting further underscores these platform dynamics: Van den Abeele et al. (2026) found that when momfluencers explicitly explained their mindful sharenting practices (e.g., not showing the child’s full face), followers responded with greater empathy and engagement, indicating that norms around child protection are actively negotiated even within commercialized sharenting.

The reliability of the SBS was satisfactory: alpha and omega ranged from 0.72–0.89 across subscales (total alpha = 0.91). Four subscales showed AVE below 0.50 (F4 = 0.35, F1 = 0.43, F5 = 0.43, F3 = 0.49). However, CR exceeded 0.70 for all, indicating sufficient convergent validity for research purposes (Fornell & Larcker, 1981; Hair et al., 2018). One F1 item (Item 4) showed a primary ML loading marginally below the 0.40 criterion (0.34). However, its CFA loading was adequate (0.63), and it captures social comparison, a conceptually important facet of sharenting motivation.

These below-threshold AVE values are consistent with the conceptual breadth of the subscales. F1 (Reinforcements and Awareness) comprises several reinforcement drivers, including advertising, social comparison, popularity-seeking, identity, and support-seeking. F3 (Social and Emotional Motives) comprises social-connection and emotional motives. F5 (Conflict) comprises different sources of tension such as partner disagreement, external criticism, and felt pressure. F4 (Awareness and Consequences) comprises distinct risk domains, including legal, privacy, and reputational concerns. In each case, the items share a common construct while retaining item-specific variance, which lowers AVE. Given the high composite reliability of these subscales, this pattern reflects their multidimensional content (Hair et al., 2018).

Exploratory analyses further indicated that active sharenting varied modestly with parental age (*η*^2^ ≤ 0.03), following a non-monotonic pattern in which parents aged 36–55 years reported the least frequent sharing, whereas risk awareness (F4) remained stable across age groups. This suggests that parental age is associated with the intensity rather than the risk-appraisal dimension of sharenting.

### 4.2. Theoretical and Practical Implications

The present findings can be interpreted through the two theoretical frameworks that motivated the development of the scale. The privacy/openness paradox (Chalklen & Anderson, 2017) is reflected in the structural position of F4: parents’ awareness of risks and consequences forms a dimension distinct from, and negatively related to, their active sharing, mirroring the tension between openness and privacy protection that the paradox describes. Self-presentation theory (Holiday et al., 2022) is reflected in the reinforcement and social-motive dimensions (F1, F3), which capture the identity-construction and impression-management functions of sharenting (i.e., parents curating a family image and seeking validation from online audiences). Given that both cognitive-evaluative and motivational-social dimensions emerged as separable factors supports the view that sharenting is a genuinely multidimensional behavior spanning both frameworks.

The SBS fills an important gap in the assessment of digital parenting behavior. Unlike the SES (Romero-Rodriguez et al., 2022), the SBS captures the motivational, cognitive, emotional, and relational dimensions of sharenting, enabling richer theoretical modeling. The concurrent validity evidence is consistent with theorized relationships: SBS total and active-sharenting subscales correlated positively with FoMO (*r*-values = 0.34–0.42), social presence (*r*-values = 0.30–0.43), and social media addiction (*r*-values = 0.24–0.34). These findings align with the view that sharenting is embedded within broader social media engagement, driven by fear of exclusion and social validation-seeking (Ní Bhroin et al., 2022). Critically, all criterion-validity correlations were in the theoretically expected positive direction: parents who post more frequently about their children score higher on the active-sharenting subscales (F2 *r* = 0.45; F3 *r* = 0.35; F1 *r* = 0.26). The negative F4 x posting frequency association (*r* = −0.17) is also theoretically coherent: more aware parents post less frequently.

The measurement invariance analyses established full scalar invariance across gender (ΔCFI = 0.005), supporting the use of the SBS for comparing factor loadings, item intercepts, and latent means between male and female parents. From a practice perspective, the SBS subscale profiles enable targeted intervention by identifying which dimension drives an individual parent’s sharenting. A profile dominated by social/emotional motives (F3) suggests interventions that address the underlying needs for social connection and validation. Low risk awareness (low F4) points to psychoeducation on the legal, privacy, and reputational consequences of sharenting (Williams-Ceci et al., 2021; Ferrara et al., 2024). Elevated conflict (F5) indicates a need for family-level communication and boundary-setting support. Finally, uncritical trust in platforms (F6) suggests digital-literacy interventions targeting realistic appraisal of platform data practices. Rather than delivering a single generic message, the SBS allows practitioners to match the intervention to the parent’s specific motivational and cognitive profile.

### 4.3. Limitations and Future Directions

The present study has several limitations. First, the sample was drawn from a single country (Türkiye) and was relatively homogeneous in sociodemographic background, which may limit the generalizability of the findings to broader and more diverse parent populations and to non-Turkish cultural contexts. Moreover, information on participants’ socio-economic status and geographic distribution (e.g., urban versus rural residence) was not collected. Evidence on how socio-economic factors relate to sharenting is limited and mixed. Some studies report that indicators such as education level are associated with sharenting frequency (Ögel-Balaban, 2021), whereas others find no such demographic effects (Ferrara et al., 2024). Together with the well-documented urban–rural disparities in internet access and online participation, this suggests that the absence of socio-economic and geographic information limits the characterization of the sample and should be addressed in future research to clarify how these factors shape sharenting across more heterogeneous populations. This relative homogeneity may also have compressed the range of active-sharenting behaviors, contributing to floor effects on several F1, F3, F5, and F6 items—particularly Item 2 (advertising-motivated sharing, M = 1.15) and Item 3 (show-off sharing, M = 1.30). Future studies should validate the SBS in more diverse cultural and socioeconomic settings.

Second, although the split-half cross-validation design avoids the common methodological flaw of using the same sample for both EFA and CFA, each half-sample (*n* = 684) is smaller than the full dataset. Future research should cross-validate the SBS structure in an entirely independent sample. Third, the study is cross-sectional, precluding inference about temporal stability. Test–retest reliability should be examined in future research. Fourth, the present study relied on self-report, which some may view as a limitation but is appropriate for assessing parents’ motives, cognitions, and attitudes toward sharenting. Future research could complement self-report with behavioral or observational indicators (e.g., actual posting records) to capture the enacted dimension of sharenting alongside its motivational basis.

Fifth, some psychometric features of the SBS warrant acknowledgement. Four subscales (F1, F3, F4, F5) yielded AVE values below the conventional 0.50 threshold, although their composite reliabilities remained adequate (all CR-values > 0.70), indicating that these subscales capture conceptually broad but internally consistent content. In addition, one item (Item 4) loaded marginally below the 0.40 primary-loading criterion but was retained on theoretical grounds. Future refinements of the scale could re-examine this item in independent samples. Finally, although parental age was examined, the age of the child was not recorded. Because sharenting practices may change as children grow older and begin to assert their own privacy preferences, future research should collect child age to test whether sharenting declines across childhood and adolescence.

## 5. Conclusions

The development and validation of the Sharenting Behavior Scale (SBS) provides an important psychometric instrument for comprehensively assessing the multifaceted factors influencing sharenting practices among parents. Using independent split-half samples for EFA (*n* = 684) and CFA (*n* = 684), the SBS demonstrated a theoretically grounded six-factor structure, excellent CFA fit (CFI = 0.956, RMSEA = 0.037), adequate-to-good reliability (alpha = 0.72–0.89), and full scalar measurement invariance across gender (ΔCFI = 0.005), permitting latent mean comparisons between male and female parents. The criterion-validity evidence confirms that higher SBS active-sharenting scores are associated with more frequent child-related posting (F2 *r* = 0.45; F3 *r* = 0.35), as theoretically expected. The finding that F4 (Awareness and Consequences) operates as a protective cognitive dimension (negatively associated with posting frequency and addictive social media use) is a theoretically novel contribution with implications for total-score interpretation and intervention design. Overall, the SBS offers a validated, multidimensional instrument that can support research on sharenting behavior, inform parental guidance programs, and underpin policy development aimed at protecting children’s online privacy.

## Figures and Tables

**Table 1:** Participants’ demographic characteristics.

Variable					
Gender	Females	Males			
*n*	747	736			
%	50.4	49.6			
Age (in years)	18–25	26–35	36–45	46–55	56–65
*n*	10	187	619	447	220
%	0.7	12.6	41.7	30.1	14.8
Marital status	Single	Married	In relationship	Prefer not to say	
*n*	101	1362	7	13	
%	6.8	91.8	0.5	0.9	
Number of children	1	2	3	4 or more	
*n*	669	630	154	30	
%	45.1	42.5	10.4	2	

**Table 2:** Distribution of participant responses on social media use and sharenting variables.

		*n*	%
Social media	*WhatsApp*	1360	91.7
	*Instagram*	980	66.1
	*YouTube*	914	61.6
	*Twitter/X*	670	45.2
	*Facebook*	663	44.7
	*LinkedIn*	447	30.1
	*Other*	85	5.7
	*TikTok*	30	2.0
	Non-user	23	1.6
Time spent on the internet daily	Less than 1 h	94	6.3
	1–3 h	721	48.6
	4–6 h	557	37.6
	More than 7 h	111	7.5
Time spent on social media daily	Less than 1 h	592	39.9
	1–3 h	828	55.8
	4–6 h	58	3.9
	More than 7 h	5	0.3
Sharing frequency	Most days	48	3.2
	1–3 times a week	68	4.6
	1–3 times a month	200	13.5
	Once a year/A few times a year	433	29.2
	Never	734	49.5
Sharing topics	Special occasions	1040	70.2
	Family holidays/travel	622	41.9
	My child’s achievements	568	38.3
	Daily life	263	17.7
	My child’s interests	185	12.5
	Other	42	2.8
Who the child is with in posts	With family members	1238	83.5
	Alone	448	30.2
	With friends	407	27.4
	In a group	173	11.7
Type of posts	Photos	1298	87.5
	Stories	395	26.6
	Videos	275	18.5
	Texts (such as memories, anecdotes, diaries, advice)	161	10.9

**Table 3:** Descriptive statistics for the 30-item SBS.

Item	M (SD)	Skewness	Kurtosis
F1: Reinforcements and Awareness			
Item 1 *	4.71 (0.61)	−2.37	5.83
Item 2	1.15 (0.43)	2.87	7.74
Item 3	1.30 (0.59)	1.79	2.05
Item 4	1.54 (0.78)	1.31	0.86
Item 5	1.42 (0.69)	1.55	1.59
Item 6	1.44 (0.70)	1.57	1.91
Item 7	1.39 (0.63)	1.36	0.68
F2: Motives and Risk Awareness			
Item 8	2.04 (1.14)	0.75	−0.60
Item 9	2.05 (1.16)	0.72	−0.73
Item 10	2.06 (1.17)	0.70	−0.81
Item 11	2.14 (1.17)	0.58	−0.90
Item 12	2.40 (1.23)	0.21	−1.28
Item 13	2.07 (1.13)	0.60	−0.92
F3: Social and Emotional Motives			
Item 14	1.76 (0.95)	1.01	0.05
Item 15	1.85 (0.96)	0.80	−0.45
Item 16	2.25 (1.12)	0.34	−1.11
Item 17	1.95 (1.03)	0.68	−0.73
Item 18	1.85 (0.98)	0.86	−0.32
F4: Awareness and Consequences			
Item 19	3.37 (1.17)	−0.34	−0.70
Item 20	3.85 (1.17)	−0.93	0.13
Item 21	4.12 (0.96)	−1.27	1.61
Item 22	3.91 (0.98)	−1.09	1.18
Item 23	3.83 (1.12)	−0.91	0.23
F5: Conflict			
Item 24	1.78 (1.02)	1.24	0.74
Item 25	1.55 (0.79)	1.17	0.22
Item 26	1.93 (1.08)	0.85	−0.31
Item 27	1.59 (0.86)	1.32	0.73
F6: Trust in Social Media			
Item 28	1.86 (0.99)	0.93	0.07
Item 29	1.82 (1.07)	1.22	0.75
Item 30	1.93 (0.98)	0.76	−0.27

* Item 1 is reverse-scored.

**Table 4:** Eigenvalues and variance explained by the six-factor ML EFA solution.

Factor	Initial EV	%	Cum %	Extraction SS	%	Cum %
1	9.16	30.53	30.53	8.63	28.78	28.78
2	2.97	9.90	40.43	2.51	8.36	37.14
3	2.08	6.92	47.35	1.48	4.92	42.06
4	1.48	4.94	52.29	1.00	3.35	45.41
5	1.11	3.69	55.98	0.69	2.30	47.71
6	1.08	3.59	59.57	0.57	1.89	49.60

**Table 5:** Factor loadings and CFA standardized estimates for the SBS.

Item	F1	F2	F3	F4	F5	F6	h2	CFA β
F1: Reinforcements and Awareness								
Item 1 *	−0.558	0.154	−0.018	0.178	−0.073	−0.070	0.378	0.516
Item 2	0.703	−0.034	0.004	0.008	0.033	0.017	0.497	0.585
Item 3	0.554	0.231	0.040	0.005	0.083	−0.031	0.370	0.699
Item 4 ^a^	0.344	0.081	0.211	−0.027	0.078	0.002	0.176	0.631
Item 5	0.425	0.232	0.100	0.021	0.089	0.016	0.253	0.724
Item 6	0.425	0.212	0.076	−0.008	0.129	0.118	0.262	0.659
Item 7	0.468	−0.018	0.287	−0.047	0.111	0.053	0.319	0.719
F2: Motives and Risk Awareness								
Item 8	0.008	0.562	0.023	0.023	0.102	0.109	0.339	0.620
Item 9	−0.018	0.674	0.037	0.026	0.006	0.076	0.463	0.734
Item 10	−0.014	0.686	−0.023	−0.045	0.044	0.153	0.498	0.786
Item 11	0.047	0.809	−0.047	−0.018	0.039	−0.017	0.661	0.794
Item 12	−0.030	0.817	0.066	−0.032	−0.084	0.014	0.681	0.831
Item 13	0.046	0.756	0.078	−0.012	−0.010	−0.057	0.584	0.770
F3: Social and Emotional Motives								
Item 14	0.082	−0.049	0.826	0.018	−0.017	0.029	0.692	0.793
Item 15	−0.054	0.190	0.522	−0.101	0.064	0.090	0.334	0.729
Item 16	0.062	0.265	0.421	0.050	−0.070	−0.071	0.264	0.507
Item 17	−0.059	0.017	0.725	−0.008	0.103	0.034	0.541	0.755
Item 18	0.083	0.131	0.586	−0.041	0.004	0.035	0.370	0.744
F4: Awareness and Consequences								
Item 19	0.064	−0.059	−0.040	0.614	−0.055	0.155	0.414	0.561
Item 20	−0.185	0.062	0.096	0.419	0.049	−0.124	0.241	0.481
Item 21	−0.140	−0.077	0.006	0.566	0.033	−0.102	0.358	0.643
Item 22	−0.008	−0.017	0.092	0.557	−0.139	0.059	0.342	0.524
Item 23	0.032	0.020	−0.057	0.695	0.055	−0.071	0.496	0.710
F5: Conflict								
Item 24	−0.022	−0.004	0.018	−0.006	0.615	0.015	0.380	0.630
Item 25	0.144	−0.020	−0.068	0.035	0.623	0.069	0.419	0.687
Item 26	−0.059	0.001	0.112	−0.018	0.652	−0.057	0.444	0.625
Item 27	0.048	0.002	−0.000	−0.031	0.642	−0.003	0.415	0.708
F6: Trust in Social Media								
Item 28	0.028	0.159	0.026	−0.011	−0.027	0.626	0.419	0.775
Item 29	0.048	−0.007	−0.030	0.032	0.020	0.703	0.499	0.633
Item 30	−0.050	−0.005	0.090	−0.040	0.011	0.719	0.529	0.755

*Note. n* = 684 (EFA subsample, seed = 2023) for EFA loadings; *n* = 684 (CFA subsample) for CFA β. F1–F6 = ML EFA oblimin pattern matrix columns arranged by theoretical subscale assignment. *h*^2^ = communality; CFA β = standardized estimate from the six-factor correlated CFA (robust maximum likelihood). * Item 1 is reverse-scored. ^a^ Item 4 loaded below the 0.40 retention criterion but was retained on theoretical grounds (its CFA loading was adequate).

**Table 6:** CFA competing model fit indices.

Model	χ^2^	Df	CFI	TLI	RMSEA	SRMR
One-factor	2736.16	405	0.664	0.639	0.099	0.095
Four-factor correlated	2055.72	399	0.767	0.746	0.083	0.092
Six-factor correlated	708.06	390	0.956	0.950	0.037	0.050
Higher-order (6 → SBS)	874.80	399	0.934	0.928	0.044	0.064

**Table 7:** Reliability and validity statistics for the SBS measurement model.

Subscale	Items	Alpha	Omega	CR	AVE	Sqrt (AVE)
F1 Reinforcements & Awareness	7	0.83	0.83	0.84	0.43	0.66
F2 Motives & Risk Awareness	6	0.89	0.90	0.89	0.58	0.76
F3 Social & Emotional Motives	5	0.83	0.84	0.84	0.49	0.70
F4 Awareness & Consequences	5	0.72	0.72	0.72	0.35	0.59
F5 Conflict	4	0.74	0.74	0.76	0.43	0.66
F6 Trust in Social Media	3	0.76	0.76	0.77	0.52	0.72
SBS Total	30	0.91	0.92	—	—	—

*Note*. CR, AVE, and √AVE are computed at the subscale level and are therefore not applicable (—) to the total score.

**Table 8:** Pearson correlations among SBS subscales and total score.

Subscale	F1	F2	F3	F4	F5	F6
F1 Reinforcements & Awareness	1					
F2 Motives & Risk Awareness	0.50 **	1				
F3 Social & Emotional Motives	0.62 **	0.64 **	1			
F4 Awareness & Consequences	0.36 **	0.26 **	0.22 **	1		
F5 Conflict	0.53 **	0.16 **	0.35 **	0.20 **	1	
F6 Trust in Social Media	0.44 **	0.53 **	0.47 **	0.21 **	0.16 **	1
SBS Total	0.80 **	0.81 **	0.81 **	−0.54 **	0.51 **	0.65 **

** *p* < 0.01 (two-tailed).

**Table 9:** Heterotrait–monotrait (HTMT) ratios among SBS subscales.

Subscale	F1	F2	F3	F4	F5	F6
F1 Reinforcements & Awareness	-					
F2 Motives & Risk Awareness	0.55	-				
F3 Social & Emotional Motives	0.75	0.72	-			
F4 Awareness & Consequences	0.47	0.33	0.31	-		
F5 Conflict	0.69	0.22	0.45	0.26	-	
F6 Trust in Social Media	0.53	0.62	0.57	0.33	0.24	-

**Table 10:** Concurrent and criterion-related validity correlations.

Scale	FoMO *r*	Social Presence *r*	SM Addiction *r*	Post Frequency *r*	Daily SM Time *r*	M (SD)
SBS Total	0.42 **	0.37 **	0.31 **	0.43 **	0.16 **	2.11 (0.43)
F1 Reinforcements & Awareness	0.40 **	0.29 **	0.34 **	0.26 **	0.09 **	1.36 (0.45)
F2 Motives & Risk Awareness	0.34 **	0.32 **	0.24 **	0.45 **	0.19 **	2.12 (0.94)
F3 Social & Emotional Motives	0.38 **	0.43 **	0.33 **	0.35 **	0.12 **	1.93 (0.78)
F4 Awareness & Consequences	−0.14 **	−0.06	−0.10 **	−0.17 **	−0.06 *	3.81 (0.75)
F5 Conflict	0.30 **	0.17 **	0.27 **	0.08 **	0.07 **	1.72 (0.71)
F6 Trust in Social Media	0.19 **	0.27 **	0.12 **	0.31 **	0.05	1.86 (0.83)

*Note.* ** *p* < 0.01; * *p* < 0.05; two-tailed.

**Table 11:** Measurement Invariance Across Gender.

Model	χ^2^	df	CFI	TLI	RMSEA	ΔCFI
Configural	1585.55	780	0.951	0.945	0.039	—
Metric	1631.25	804	0.949	0.945	0.039	0.001
Scalar	1744.61	828	0.944	0.941	0.040	0.005

*Note.* ΔCFI represents the change in CFI relative to the preceding nested model; it is not applicable for the configural model, which serves as the baseline. Values of ΔCFI ≤ 0.01 indicate invariance (Cheung & Rensvold, 2002).

**Table 12:** Sharenting scores by parental age group.

Measure	18–25 Years	26–35 Years	36–45 Years	46–55 Years	56+ Years	*F*	*η* ^2^
Posting frequency	2.50 (2.12)	2.08 (1.03)	1.80 (0.95)	1.65 (0.96)	2.12 (1.40)	9.67 **	0.027
SBS total	2.23 (0.59)	1.99 (0.54)	1.83 (0.49)	1.77 (0.51)	1.90 (0.50)	7.62 **	0.022
F1 Reinforcements & Awareness	1.77 (0.73)	1.45 (0.50)	1.33 (0.42)	1.33 (0.44)	1.44 (0.49)	6.59 **	0.018
F2 Motives & Risk Awareness	2.43 (0.83)	2.40 (1.02)	2.16 (0.95)	2.00 (0.92)	2.01 (0.83)	7.08 **	0.020
F3 Social & Emotional Motives	2.26 (0.80)	2.15 (0.86)	1.89 (0.77)	1.83 (0.75)	2.02 (0.76)	6.88 **	0.019
F4 Awareness & Consequences	3.66 (0.52)	3.74 (0.71)	3.83 (0.75)	3.87 (0.77)	3.74 (0.75)	1.59	0.005
F5 Conflict	2.25 (0.73)	1.76 (0.71)	1.62 (0.67)	1.71 (0.71)	1.94 (0.75)	9.71 **	0.027
F6 Trust in Social Media	2.67 (0.98)	1.94 (0.82)	1.83 (0.83)	1.82 (0.83)	1.92 (0.82)	3.53 **	0.010

*Note.* Values are M (SD). Posting frequency is scored so that higher values indicate more frequent sharing (5 = most days, 1 = never). *F* and *η*^2^ are from one-way ANOVAs (df = 4; denominator 1363–1415 due to listwise deletion). The 18–25 group (*n* = 10) is reported for completeness. ** *p* < 0.01.

## Data Availability

The data are available from the corresponding author upon reasonable request.

## Appendix A. Sharenting Behavior Scale

All 30 SBS items, grouped by subscale. Response scale: 1 (Definitely Disagree) to 5 (Definitely Agree). (R) = reverse-scored (6 minus x). Items 19–23 (F4) are scored in their stated direction for subscale analyses and reverse-scored when computing the total SBS score.

**F1: Reinforcements and Awareness**

1. (R) I am careful not to share sensitive information or images of my child on social media.
2. I share things about my child on social media for advertising and marketing purposes.
3. I post pictures of my child to show off or get compliments from my friends.
4. I like to see how my parenting practices compare to other parents on social media.
5. I believe that sharing things about my child on social media makes me more popular or interesting.
6. Sharing things about my child on social media is an important part of my parenting identity.
7. I share things about my child on social media to receive or seek emotional support from my online community.

**F2: Motives and Risk Awareness**

8. I am aware of the potential privacy risks associated with sharing things about my child on social media, but I still do it.
9. I value my child’s privacy, but I also enjoy sharing things about them on social media.
10. I like to share my feelings about my child on social media, such as how proud I am of them or how much I love them.
11. I share things about my child on social media because it makes me feel proud.
12. I share things about my child on social media because it makes me happy.
13. I share things about my child on social media because it excites me.

**F3: Social and Emotional Motives**

14. I feel that sharing things about my child on social media is a way to connect with other parents.
15. I think that sharing personal stories about my child on social media helps to build connections and foster empathy.
16. I believe that social media can provide a valuable source of emotional support for parents.
17. Sharing things about my child on social media helps me connect with other parents who share similar experiences.
18. I believe that sharenting practices are a way to connect with others who share similar cultural or community backgrounds.

**F4: Awareness and Consequences**

19. I am aware of the legal implications of sharing things about my child on social media.
20. I think about how my sharenting practices might affect my child’s future digital footprint and reputation.
21. I am aware of the potential risks of sharing things about my child on social media.
22. I am aware of the benefits and drawbacks of using technology and social media for parenting purposes.
23. I am aware of the potential consequences of sharing things about my child on social media for advertising and marketing purposes.

**F5: Conflict**

24. I have had disagreements with my partner or other family members about sharing things about our child on social media.
25. I have received negative comments or criticism about sharing things about my child on social media.
26. My friends or family members have expressed concerns or objections about my sharing of things about my child on social media.
27. I feel pressure from family or friends to share less things about my child on social media than I am comfortable with.

**F6: Trust in Social Media**

28. I trust social media platforms to keep the things that I share about my child secure.
29. I believe that social media platforms have my child’s best interests in mind.
30. I trust social media platforms to enforce their policies regarding my child’s privacy.
